# Precision Diagnosis in Cutaneous Head and Neck Squamous Cell Carcinoma †

**DOI:** 10.3390/biomedicines14030556

**Published:** 2026-02-28

**Authors:** Ameya A. Asarkar, Nrusheel Kattar, Karthik N. Rao, Alessandra Rinaldo, M. P. Sreeram, Eelco de Bree, Juan Pablo Rodrigo, Carlos M. Chiesa-Estomba, Orlando Guntinas-Lichius, Ashok R. Shaha, Alfio Ferlito

**Affiliations:** 1 Department of Otolaryngology/Head and Neck Surgery, LSU Health Sciences Center, Shreveport, LA 71103, USA; nrusheel.kattar@lsuhs.edu; 2 Department of Head and Neck Oncology, Sri Shankara Cancer Foundation, Bangalore 560004, India; karthik.nag.rao@gmail.com (K.N.R.); drsreeram111@gmail.com (M.P.S.); 3 ENT Unit, Policlinico Città di Udine, 33100 Udine, Italy; dottalerinaldo@gmail.com; 4 Department of Surgical Oncology, Medical School of Crete University Hospital, 71110 Heraklion, Greece; debree@uoc.gr; 5 Servicio de Otorrinolaringología, Hospital Universitario Central de Asturias, 33011 Oviedo, Spain; 6 Instituto Universitario de Oncología del Principado de Asturias, Instituto de Investigación Sanitaria del Principado de Asturias, Universidad de Oviedo, CIBERONC, ISCIII, 33011 Oviedo, Spain; 7 Department of Otolaryngology–Head and Neck Surgery, Donostia University Hospital, 20014 San Sebastián, Spain; chiesaestomba86@gmail.com; 8 Faculty of Medicine, Deusto University, 48007 Bilbao, Spain; 9 Department of Otorhinolaryngology, Jena University Hospital, Am Klinikum 1, D-07747 Jena, Germany; orlando.guntinas@med.uni-jena.de; 10 Head and Neck Service, Memorial Sloan Kettering Cancer Center, New York, NY 10065, USA; shahaa@mskcc.org; 11 International Head and Neck Scientific Group, 35100 Padua, Italy; profalfioferlito@gmail.com

**Keywords:** skin neoplasms, non-melanoma skin cancer, early detection, liquid biopsy, biomarkers, radiomics, gene expression, dermoscopy

## Abstract

Precision oncology has been evolving rapidly, with increasing emphasis on early detection and personalized diagnostic approaches that translate into tailored treatment algorithms. The integration of molecular markers, quantitative imaging approaches and artificial intelligence (AI) in the diagnostic workflow of cutaneous squamous cell carcinoma (cSCC) has increased accuracy and has the potential to improve early detection rates in these cancers. Sun exposure is the primary etiologic factor in the development of cSCC. The primary objective of this review is to evaluate the current state and future directions of modalities and practices in diagnostic techniques for cSCC. Specifically, this review summarizes the key genetic alterations and potential molecular targets in cSCC. High-risk genetic mutations and pathways implicated in the pathogenesis of cSCC include p53, NOTCH, RAS/MAPK, cell-cycle, and adhesion pathways. This review further explores current and emerging modalities in optical imaging techniques and molecular-based diagnostic modalities in cSCC. Further, we discuss the role of radiomics and AI in the diagnostic work-up of cSCC. These techniques have the potential to enable more accurate risk models that refine conventional histopathology and guide personalized interventions. However, there are limitations to the clinical application of several of these modalities, with cost being an important driver. These challenges have been discussed in detail within this review. Nevertheless, ongoing research is focused on improving the workflow and initiating a shift in clinical practice with application of precision diagnostics as a standard of care.

## 1. Introduction

Cutaneous squamous cell carcinoma (cSCC) is the second most common type of skin cancer, with sun-exposed areas of the head and neck region being the most affected anatomical sites [1,2,3]. Although most cSCCs have good oncologic outcomes, the prognosis is poor in high-risk cSCC, as these frequently present in metastasis or in an unresectable stage [4]. Early detection of high-risk subtypes is the cornerstone for improving outcomes and survival rates. Specifically, cutaneous head and neck squamous cell carcinomas (cHNSCCs) tend to be more aggressive, with local and distant recurrence rates between 5% and 20%. On the contrary, cSCCs on the extremities have been reported to have local recurrence and metastasis rates of 3% and 2.5%, respectively [5,6]. Further, surgical excision or definitive radiotherapy for cHNSCC is challenging given the anatomical restraints and impact on cosmetic outcomes [7].

There is significant overlap of the pathogenesis and risk factors of cSCC across different anatomical sites, as the central causative factor remains persistent sun exposure [2]. Traditional diagnostic and prognostic tools in cSCC include clinical examination, imaging and histopathology. Dermatoscopy is a rapid, noninvasive modality to reliably screen cutaneous lesions [8]. However, these are prone to diagnostic errors and are dependent on the clinician’s experience and abilities [9]. Staging systems such as the American Joint Commission on Cancer (AJCC) 8th edition and the Brigham and Women’s Hospital (BWH) classification are susceptible to interobserver variability and have limitations with the classification of these tumors in several patients [10,11]. Further, there is variability between the AJCC and BWH classifications, with BWH showing a higher specificity and positive predictive value (PPV) in identifying the risk of metastasis [10]. Thus, there is a need for refining these staging systems by incorporating the predictive capabilities of the high-risk features of cSCC. These high-risk factors in cSCC including tumor size, depth of invasion, perineural invasion, differentiation and immunosuppression, which are corelated with poor prognosis [12]. However, studies have shown heterogeneity in the prognostic ability while utilizing these factors alone. This has led to an increased integration of biologic molecular markers and quantitative imaging including radiomics, artificial intelligence (AI) and machine learning algorithms to further refine the diagnostic and prognostic accuracy of these techniques as adjuncts to traditional risk assessment variables.

Recent literature reports support the integration of some of the genetic and molecular tests in the management of cSCC. Specifically, studies have shown promising results with the use of commercially available gene expression profiling (GEP) assays. These assays have successfully stratified lymph nodal metastasis risk independently from the traditional pathologic variables [13]. Other tumor tissue-based molecular approaches include the determination of tumor mutational burden (TMB) and PDL-1 expression [14]. A liquid biopsy-based approach is additionally emerging as a diagnostic tool and includes proteomic plasma profiling (PPP), circulating microRNA (miRNA), circulating tumor DNA (ctDNA) and circulating tumor cells (CTCs) [15,16,17]. The latter approaches have a distinct advantage in cancer surveillance, thus playing a real-time role in detecting recurrences earlier than the traditional imaging modalities.

**Figure 1:**
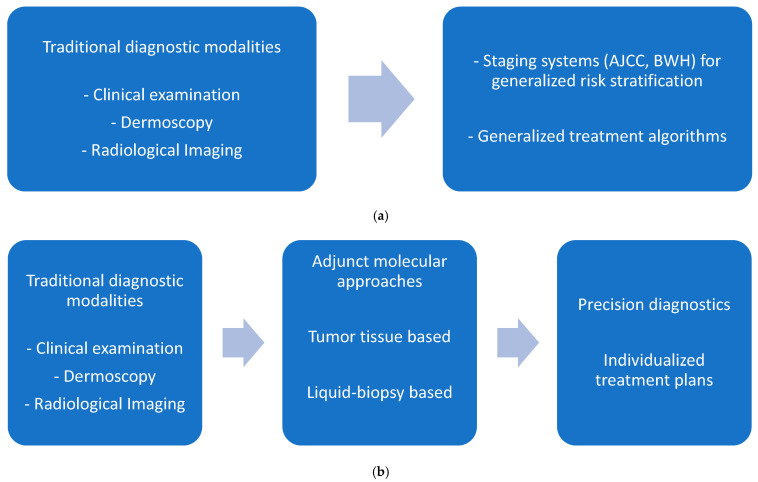
(**a**) Traditional diagnostic workflow in cSCC. (**b**) Workflow for precision diagnosis in cSCC.

This review will summarize the mutational landscape of cSCC and then discuss the current and emerging diagnostic standards in cSCC, including advances in optical imaging techniques such as reflectance confocal microscopy (RCM), multiphoton microscopy (MPM), molecular tests and the integration of AI in the diagnostic algorithms of cSCC. When available, data specifically for the head and neck region will be discussed. By synthesizing the available literature, this review provides the current state of diagnostic modalities in cSCC, focusing on areas of future research for precision oncology (Figure 1).

## 2. Molecular Biology and Pathology of cSCC

cSCC is defined by an ultraviolet (UV)-driven, highly mutated genome in which recurrent disruption of the p53, NOTCH, RAS/MAPK, cell-cycle, and adhesion pathways intersects with an immunomodulatory microenvironment and histologic differentiation state to determine clinical behavior [18,19]. Integrating these genetic and microenvironmental features with refined grading systems improves risk stratification and underpins emerging precision diagnostic approaches in cSCC [18,20] (Table 1).

### 2.1. Key Genetic Alterations

Whole-exome and meta-analytic sequencing consistently identify *TP53* and *NOTCH1/2* as the dominant driver events in cSCC, with loss-of-function mutations in the p53 and NOTCH pathways present in roughly two-thirds to four-fifths of tumors [18,21]. *TP53* mutations often arise early in actinic keratoses and in chronically sun-exposed “field cancerization” skin, establishing a background of genomically unstable keratinocytes from which invasive cSCC emerges [18]. *CDKN2A* mutation is a frequent cooperating alteration that inactivates both p16INK4a and p14ARF, converging with TP53 loss to disable the G1/S checkpoint and apoptosis [18,19]. Activating *HRAS* mutations and other Ras/MAPK/PI3K alterations (e.g., in PIK3CA, MTOR, PTEN) are seen in a minority of tumors but mark proliferative signaling-addicted subsets, often in higher-risk disease [18,19].

*FAT1* truncating mutations are recurrent in cSCC and other squamous malignancies, and they promote a hybrid epithelial–mesenchymal (EMT) state with enhanced stemness, YAP-driven transcription, and metastatic competence [22,23]. Large-scale analyses also highlight frequent alterations in chromatin remodeling and DNA damage response genes (e.g., *KMT2D*, *EP300*, *PBRM1*, *BRCA2*), which are enriched in metastatic or high-risk tumors and may signify particularly unstable genomes [18,19]. While classic mismatch-repair-deficient cSCC is rare, broader DNA repair defects and distinctive signatures are emerging as contributors to the biology of metastatic cSCC and potential predictors of immunotherapy sensitivity [24].

### 2.2. Tumor Immune Microenvironment

cSCC typically exhibits very high TMB, yet immune control is variably effective and conditioned by tumor differentiation state and the local stromal context [18,19]. Early, well-differentiated (epithelial) cSCCs tend to show relatively modest T-cell infiltration, with PD-L1 expression on tumor cells as a dominant immune-evasion mechanism, whereas more mesenchymal or poorly differentiated lesions display dense infiltrates of exhausted CD8+ T cells and NK cells together with the expression of multiple inhibitory ligands (PD-L1, CD80, CD155) linked to the PD-1, CTLA-4, and TIGIT pathways [18,19,25,26]. FOXP3+ regulatory T cells are highly enriched in cSCC relative to normal skin, often comprising a large fraction of intratumoral T cells and contributing to local immunosuppression via IL-10, TGF-β, and ectonucleotidase activity [27,28].

The innate immune compartment reinforces immunosuppression in cSCC through multiple coordinated mechanisms. Tumor-associated macrophages adopt a mixed M1/M2 state that promotes invasion via cathepsin K, while dendritic cells show impaired T-cell activation due to TGF-β and IL-10 [29]. Myeloid populations, including tumor-associated macrophages, immature dendritic cells, and myeloid-derived suppressor cells, cluster with EMT-programmed tumor cells and cancer-associated fibroblasts at invasive fronts to reinforce immunosuppression and matrix remodeling [30,31]. Downregulation of vascular E-selectin on tumor-associated endothelium further impairs the recruitment of skin-homing effector T cells, favoring the accumulation of Tregs and other suppressive subsets despite a high neoantigen load [24]. These findings support a model in which EMT plasticity in cancer cells shapes the dominant checkpoint axis (PD-1/PD-L1 versus CTLA-4/TIGIT) and thereby influences which immunotherapy is most effective in individual tumors [19,32]. The mutational landscape of the tumor immune microenvironment is summarized in Table 1.

### 2.3. Pathologic Grading and Biomolecular Correlates

Traditional Broders grading, based largely on percent keratinization, captures broad differentiation categories but suffers from interobserver variability and limited alignment with molecular risk features in cSCC [31,33]. Multifactorial systems derived from Bryne’s criteria, which score keratinization, nuclear pleomorphism, mitotic activity, and inflammatory response at the invasive front, correlate more tightly with proliferative and genomic markers such as Ki-67, p53 accumulation, and patterns of perineural or lymphovascular invasion [31,33]. To enable accurate prognostic classification and guide the appropriate management of cSCC, pathology reports should document several well-established high-risk features. These include histologic subtype (acantholytic, spindle cell, verrucous, or desmoplastic variants), degree of differentiation (well-differentiated, moderately differentiated, poorly differentiated, or undifferentiated), tumor depth measured as the maximum vertical diameter in millimeters, level of dermal invasion assessed by Clark’s classification, presence of perineural invasion, lymphovascular invasion, and margin status indicating whether surgical edges are clear or involved by tumor cells [34]. These parameters collectively inform risk stratification, treatment decisions, and surveillance strategies for individual patients [35]. High-grade or poorly differentiated tumors show higher frequencies of *TP53*/*CDKN2A* alterations, greater copy-number complexity, and enrichment of transcriptomic signatures for EMT, focal adhesion, hypoxia, and inflammatory signaling [36].

Immunohistochemical surrogates provide additional biomolecular resolution within histologic grades. Increased Ki-67 and diffuse aberrant p53 staining are associated with higher Bryne’s scores and worse outcomes, whereas strong E-cadherin and cohesive keratinization suggest lower risk [24,33]. The integration of grading with mutational data has begun to delineate subgroups such as *FAT1*-mutant, EMT-high cSCC with stemness and metastatic propensity, and Ras/MAPK-activated tumors that are highly proliferative but variably differentiated [18,22]. Furthermore, the role of E-cadherin in cSCC metastasis has been hypothesized in several studies. Downregulation of E-cadherin is key for EMT. Thus, the under expression of E-cadherin may be helpful in predicting a higher likelihood of metastasis [37]. Table 1 summarizes the key genetic alterations and the clinical correlations of these findings in diagnostic decision-making.

As multi-omics and single-cell data mature, future grading frameworks are likely to evolve toward combined morphologic–molecular indices that incorporate driver genotype, EMT/stemness programs, and immune contexture to improve precision diagnosis and treatment selection in cSCC [19,30].

### 2.4. Epigenetics in cSCC

A critical driver in the role of epigenetics in cSCC is the interplay with certain metabolite cofactors that regulate several enzymatic reactions. Evidence suggests that PARP1-mediated DNA repair in the setting of UV-radiation is promoted by nicotinamide adenine dinucleotide (NAD+), an essential co-enzyme of redox reactions for ATP production [38,39]. Secondly, DNA methylation plays a role in the carcinogenesis of cSCC as well. A study by Li et al., showed upregulation of the maintenance DNA methyltransferase, DNMT1, in human UVB-radiation-induced cSCC, while the DNA demethylation enzymes TET1 and TET2 were downregulated, further anchoring the interplay of DNA methylation as a player in epigenetics for cSCC [40]. Similarly, histone modifiers are also implicated as frequently mutated genes in cSCC [41]. Some of the histone modifiers implicated in cSCC include CBP (*CREBBP*) complexed with p300 (*EP300*), which forms a co-activator complex that binds several transcription factors. This has been shown to suppress EGFR–Ras–ERK signaling to repress cSCC in animal studies [42].

Molecular analysis has a role in stratifying cSCC into high-risk phenotypes. Incorporating biologic phenotypes along with the well-established histopathologic classification systems would allow for better precision in identifying more aggressive subsets.

## 3. Advances in Optical Imaging Techniques in cSCC Diagnosis

### 3.1. Microscopy/Dermatoscopy

Traditional diagnostic modalities encompass clinical and dermoscopic examination, as well as subsequent microscopic confirmation of invasive cancer. Dermatoscopy particularly improves the diagnostic accuracy compared to the unaided eye [8]. It is rapid, cost-effective and universally available. However, the interpretations are subjective and require extensive training in the field to reduce errors in diagnosis. Studies have reported the sensitivity and specificity of dermatoscopy to be 79% and 87%, respectively [43]. In addition to these modalities, several advanced diagnostic and prognostic aids have emerged, enhancing the accuracy and early detection of these cancers [44].

Microscopy has evolved into a more diversified toolkit to provide enhanced histologic resolution, specific contrast at the molecular level and computational augmentation for whole-slide analysis. These advances have led to reduced diagnostic delays, a decreased need for additional biopsies, improved margin assessment and molecular aiding of prognostication and treatment selection.

### 3.2. Reflectance Confocal Microscopy (RCM)

RCM is a Food and Drug Administration (FDA)-approved noninvasive optical imaging technique that uses a near-infrared coherent monochromatic light to assess skin lesions [45]. Typically, RCM employs a low-power Diode LASER emitting infrared light of 830 nanometers (nm). En face images of the epidermis and superficial dermis can be obtained to assess architectural disarray and focal invasion. RCM is particularly helpful as an adjunct to microscopy in equivocal lesions that lack characteristic dermoscopic features [45].

Although the use of RCM involves a learning curve, a recent study showed that RCM improved the reliability of histological diagnosis, thus eliminating the need for repeated biopsy [46]. The sensitivity and specificity of RCM for the diagnosis of SCC are 78–100% [47]. RCM enables the assessment of a wider area due to its larger field of view than that of traditional microscopic views [48]. RCM also has a promising role in the real-time intraoperative evaluation of surgical margins [49].

An important limitation of RCM is the maximum imaging depth that it can assess. The resolution of the images substantially decreases below 100 to 150 μm (although the maximum depth is about 300 μm). Furthermore, diagnostic accuracy with RCM is operator dependent [50].

### 3.3. Multiphoton Microscopy (MPM)

MPM utilizes autofluorescence (AF) from the skin to observe cellular structures, along with the redox status and collagen fiber quantification [51]. Biological tissues are composed of several fluorescent substances including proteins, porphyrins, purines, etc., which are excitable by photons. The excitability and emission are unique and can generate microenvironmental information from within the epidermis and dermis [52].

MPM uses longer excitation wavelengths (700–1000 nm), thus enabling the evaluation of deeper tissues compared to RCM. The deeper penetration provides intrinsic contrast for collagen, revealing tumor–stroma interfaces and detecting collagen remodeling that is typical of cSCC [53]. Further, MPM reliably characterizes invasive patterns in cSCC and perineural spread as well. The main limitations are the instrument complexity and the cost associated with its routine clinical application [53]. However, the FDA recently granted marketing clearance for a handheld MPM system that has a reliable accuracy of >90% in identifying skin lesions [54].

### 3.4. Ex Vivo Confocal Microscopy (EVCM)

EVCM is an optical imaging technique that allows microscopic examination of freshly excised unfixed tissue. A confocal microscope specifically designed for reflectance imaging is used to perform ex vivo examination of tissue. Recent studies have shown the utility of EVCM for intraoperative margin assessment, and it has a high concordance with paraffin histology. A study reported a sensitivity and specificity of 95% and 96% for the identification of SCC using EVCM, respectively [55]. At this time, EVCM cannot replace frozen sections as the gold standard for intraoperative assessment of surgical margins. However, it may have clinical application for the screening of grossly positive margins with negative margins still confirmed with frozen section [56,57]. As the EVCM techniques evolve, margin assessment times may shrink and close the diagnostic lacunae for margin control [58,59].

### 3.5. Vibrational and Label-Free Chemical Imaging

Raman spectroscopy offers a noninvasive technique that captures molecular vibrations that translate into distinct Raman spectroscopic signatures found in cSCC. Further, Raman spectroscopy and stimulated Raman scattering (SRS) microscopy delineate the biochemical composition (lipids, proteins, nucleic acids) of cancer versus normal skin [60]. Emerging studies that integrate AI with SRS have been shown to improve diagnostic accuracy by identifying molecular signatures and precise spectral markers [61,62]. This integration has the potential to develop into a tool for longitudinally monitoring molecular changes. The combination of Raman spectroscopy with other modalities, such as optical coherence tomography (OCT), ultrasound or magnetic resonance imaging, has the potential to provide both morphological and biochemical insights into cutaneous lesions [63].

Specific challenges to the practical application of Raman spectroscopy, such as temperature fluctuations and humidity, can introduce noise into Raman spectra. In addition, overexposure to LASER light can lead to sample degradation and fluorescence signal interference [63]

### 3.6. Whole-Slide Imaging (WSI) and Digital Pathology

WSI involves scanning traditional glass slides into high-resolution digital images, which can be accessed as digital images. WSI provides significant advantages by promoting multicenter collaboration and standardizing diagnostic workflows [64]. Further, WSI improves data storage, tracking and retrieval. Additionally, digital WSI can be preserved long-term with less risk of physical damage and improved accessibility [65].

The challenges to WSI are two-fold: technological barriers and regulatory issues. Investment in cloud-based platforms to host digital images and support for a high-speed network is crucial. These cloud-based platforms need to comply with data security protocols and to have safeguards for protecting sensitive patient information [66]. Standardization and integration of WSI images into the existing electronic medical records (EMRs) and laboratory information systems (LISs) will help in streamlining diagnostic workflows [67]. The integration of AI in WSI platforms has transformed the utility of digital pathology into a more robust diagnostic modality with improved diagnostic accuracy and consistency [68].

The landscape of diagnostic microscopy for cSCC now includes an array of noninvasive modalities, including RCM, MPM, EVCM Raman spectroscopy and SRS microscopy, as well as WSI and digital pathology. While these technologies have been validated in research and early clinical studies, their widespread adoption will still require multi-institutional validation with prospective clinical studies and cost-effectiveness analysis. However, with the advent of AI, these have the potential to improve precision diagnostic workflows and aid in individualized therapy for patients with cSCC. AI models integrating deep learning neural networks have enhanced the capabilities of recognizing complex patterns of histopathological changes. One strategy employed by pathologists using these technologies is to flag suspicious areas for a detailed review by the pathologist, thus improving the diagnostic yield [69].

Recently, the FDA approved the first AI-enabled device (DermaSensor) for skin cancer detection based on the optical imaging technique of elastic scattering spectroscopy [70]. This is an important step toward a greater integration of AI in screening of skin lesions for early detection and appropriate management by a specialist. DermaSensor had a sensitivity and negative predictive value (NPV) of 95.5% and 96.6%, respectively, in a large study (DERM-SUCCESS) conducted on 1579 lesions [71]. Despite this, it also had a low specificity of 20.7% in the same study, indicating that there is still the potential of increased unnecessary referrals and biopsies. A further limitation was that, of the DERM-SUCCESS trial’s population, 97.1% identified as White and 13% were categorized as Fitzpatrick V/VI (most pigmented skin types) [71]. Thus, the datasets utilized for the AI algorithms are likely to have this limitation. As real-world data are being generated with the use of DermaSensor, FDA regulators will be examining its performance in diversified population phenotypes. Regardless, this FDA approval has been a milestone for AI-enabled medical devices.

Table 2 summarizes the common optical imaging techniques.

The greatest impact of optical imaging techniques will be in identifying malignant lesions in high-risk patients, with impacts on treatment monitoring and surveillance without the need for unnecessary biopsies.

## 4. Role of Radiomics in the Management of cSCC

Radiomics encompasses the automated extraction of mathematically defined features from medical images, including ultrasound, CT scan, MRI scan, PET scan, and dermatoscopy, and subsequent characterization of individual biologic features [72,73]. In recent years, radiomics has gained traction for predictive biomarker diagnosis, assessment of treatment response and prognostication [74]. The pairing of important clinical predictors with radiomic analyses forms the basis of predictive models that accurately reflect the metabolic and morphologic phenotypes of the primary tumor and lymph nodes [75].

Studies have shown that the integration of AI has aided in the detection of skin cancer [76,77]. These automated tools can be categorized into conventional machine learning and deep learning-based models. Conventional or traditional machine learning models incorporate image processing, segmentation and extraction based on low-level radiomics approaches. Studies using these models have shown an accuracy rate of 85–96% in identifying cancerous versus non-cancerous skin lesions [78,79]. A study by Kumar et al. developed an algorithm for training an artificial neural network to identify cancerous skin lesion with an accuracy of up to 97% [80]. Alternatively, the newer branch of machine learning uses deep learning-based techniques for image processing. This differs from the traditional models by obviating the need for preprocessing, segmentation and extraction of the images for analysis [81]. The accuracy rate reported with the application of these models is 96–99% [82,83]. Although radiomics will play a vital role in precision diagnostics, the standardization of the data is still nascent due to the wide variation in imaging protocols, extraction techniques and tumor segmentation methods [84]. An important consideration is the cost effectiveness in early cSCC, as it would be difficult to justify the time-intensive imaging procedures for diagnosis in these lesions, thus limiting the role of radiomics to follow-up in response assessment or in advanced cSCC [85,86]. Furthermore, logistical issues frequently encountered include high specification hardware for data processing and storage. Cloud storage platforms could possibly circumvent these issues. Access to cloud storage would also need an uninterrupted data connection with a high bandwidth [77].

## 5. Molecular and Biological Approaches

Emerging technologies are reshaping how cSCC can be detected, risk-stratified, and monitored, moving the field toward minimally invasive, data-rich precision diagnostics. Broadly, molecular and biologic approaches are divided into liquid biopsy or tumor-based approaches. Table 2 summarizes the various liquid-based approaches that are commonly investigated in cSCC.

### 5.1. Liquid Biopsy-Based Approaches

#### 5.1.1. ctDNA and Liquid Biosignatures

ctDNA exosomes and tumor-derived extracellular vesicles enable noninvasive sampling of the mutational and epigenetic landscape of cSCC, with potential applications in early detection, minimal residual disease monitoring, and surveillance for recurrence after surgery or radiotherapy [87]. ctDNAs are short DNA fragments (130–145 base pairs). Tumor-informed assays that track patient-specific mutations can detect progression or relapse earlier than cross-sectional imaging, and nano-enabled electrochemical or plasmonic biosensors are being developed to improve sensitivity for SCC-related nucleic acids and proteins in blood, saliva, or wound exudate [88]. Currently, ctDNA is not FDA approved for the monitoring of cSCC; however, ongoing clinical trials are evaluating its efficacy as a reliable biomarker [89]. A potential barrier confounding the use of ctDNA is the co-occurrence of multiple SCCs, a commonly seen phenomenon in sun-exposed areas. The challenge would be to trace the liquid biopsy signals to the causative primary tumor [90]. Furthermore, ctDNA extraction techniques are heterogenous and include manual and automatic commercially available kits. These kits differ in their isolation principles including ones based on the interaction between DNA molecules and magnetic or silica gel membranes. These variables have a significant impact on the accuracy and reproducibility of ctDNA analysis data [91]. It is also challenging to define the ctDNA libraries for cSCC due to the scarcity of molecular data in publicly available databases, thus some specific mutations may potentially be missing compared to tissue-based testing. Thus, although ctDNA has shown promise as a reliable biomarker in certain cancers such as melanoma and Merkel cell carcinoma, its role in cSCC is still evolving, especially in low tumor burden cSCC [91,92,93].

#### 5.1.2. Proteomic Plasma Profiling

Proteomic plasma profiling (PPP) refers to the technique of analyzing proteins in plasma to identify specific biomarkers that can serve in the diagnosis and prognosis of cancer [94]. Mass spectrometry is a commonly utilized method for the process of plasma proteomics. A few studies have identified specific proteins in cSCC. Wang H. et al. investigated the utility of serum matrix metalloproteinase-13 (MMP-13) as a potential biomarker for cSCC [94]. The authors reported a sensitivity and specificity of 81.7 and 82.4%, respectively, with an area under the curve (AUC) of 0.87 (95% CI [0.78 to 0.95]). The cut-off value for MMP-13 in this study was 290 pg/mL [94]. Further, serum MMP-13 also predicted lymph node involvement with an AUC of 0.94 (95% CI [0.88 to 0.99]) and a sensitivity and specificity of 93.8 and 88.5%, respectively, for a cut-off value of 430 pg. The clinical relevance was further corroborated with a decrease in the serum MMP-13 levels following surgery in these patients. Studies have demonstrated the expression of MMP 13 in cSCC and not in normal skin. Combining ctDNA and PPP as a composite biomarker has been explored in other cancers [95]. However, cSCC was not a part of the cancer panel for this multi-analyte blood test [96].

#### 5.1.3. Circulating miRNA

miRNAs are composed of endogenous, non-coding RNAs primarily involved in controlling basal cell processes typically via post-transcriptional regulation of gene expression [97]. Several processes, such as apoptosis, invasion, cell proliferation and migration, are affected due to dysregulation in miRNA expression [98]. Studies have reported miRNAs as stable blood-based markers for cancer detection [99]. In a cohort of 79 patients Cañueto et al., reported the correlation of miRNA (miR)-203 and miR-205 expression with prognostic subgroups of cSCC. Other studies have shown that the overexpression of pre-miR-31 correlates with increased invasion in cSCC [100]. Recently, Hossain et al. validated miRNAs using qRT-PCR and in vitro phenotypic assays in relation to the progression of cSCC, thus suggesting a role of miRNA expression in keratinocyte transformation [101]. The authors thus suggested that the expression of miR-31 may be suitable as a keratinocytic cancer biomarker.

#### 5.1.4. Circulating Tumor Cells

Circulating tumor cells (CTCs) are viable tumors cells released by malignant tissues into the bloodstream at low concentrations. Detectable CTCs are correlated with adverse oncologic outcomes including recurrence and risk of metastasis [102,103]. However, data on the role of CTC in cSCC are scarce. Morosin et al. identified CTCs in 80% of cHNSCCs with concentrations up to 44 cells/9 mL blood [104]. The use of CTCs as a potential biomarker in cHNSCC requires further research into the biology of CTCs. However, this will require a more comprehensive characterization through high-throughput genomic and proteomic sequencing.

The currently studied liquid-biopsy approaches are summarized in Table 3.

Clinical trials are ongoing to investigate the role of ctDNA as a potential biomarker. Liquid biopsy could be integrated in the surveillance or treatment response paradigms, if validated.

### 5.2. Tumor Tissue-Based Approaches

Traditional standard of care diagnostic methods in cSCC are typically tissue-based. The advancements in precision diagnostics based on these methods can be relatively easily implemented when compared to the liquid biopsy-based approaches. A few of the tissue-based approaches are discussed below.

#### 5.2.1. Gene Expression Profiling

The role of GEP in cSCC has been primarily studied in the context of predicting metastatic risk. The application of the traditional staging systems of the AJCC and BWH have their limitations with predicting metastasis [10,105]. GEP captures the genetic landscape of the tumors to provide a predictive risk assessment [106]. The 40-GEP test developed for cSCC includes genes responsible for the molecular processes relevant to progression, repair and immune response. Further, machine learning algorithms were leveraged to classify tumors based on the expression of the selected specific genes [13,107]. The 40-GEP test classifies tumors into three risk categories: class 1 (low risk), class 2A (higher risk), and class 2B (highest risk). These risk categories correlate well with the metastasis-free survival rates across the three categories, with the prognostic value of GEP classification being an independent predictor [108]. This is now commercially available as a 40-GEP test (DecisionDx-SCC; Castle Biosciences, Inc., Friendswood, TX, USA) [107].

A recent systematic review and meta-analysis compared the 40-GEP test to the AJCC 8th edition and BWH staging systems and noted that the GEP classification had a significantly higher PPV for metastasis [109]. Leveraging the clinicopathologic characteristics and molecular insights strengthens the risk assessment framework. The data on the use of the 40-GEP test as a crucial component of a precision diagnostic toolkit in cHNSCC are promising; however, there are certain barriers to its widespread adoption. Accessibility due to higher costs is still a hurdle. Additionally, continuous validation of the GEP data, along with the radiational clinicopathologic characteristics, is crucial for the machine learning algorithms to improve the accuracy of risk stratification for cHNSCC. Future research on the cost-effectiveness will provide more insight into its clinical application. As the data on GEP accumulate, the GEP results are currently recommended to be used as additional data to the standard diagnostic and prognostic indicators.

#### 5.2.2. Programmed Death-Ligand 1 Expression

Programmed death-ligand 1 (PD-L1) expression has been shown to be associated with poor prognosis in several HNSCCs. High-risk pathologic features such as higher histologic grade, tumor thickness and increased metastatic risk have been seen in cSCC with PD-L1 expression [110,111,112]. The role of PD-L1 as the sole biomarker for cSCC is limited; however, it plays a role in prognostication following immunotherapy treatment [25].

Winter et al. provided a comprehensive immunohistochemical analysis of the tumor microenvironment in basal cell carcinoma (BCC) and cSCC, revealing distinct immune cell infiltration patterns that could inform precision oncology approaches for non-melanoma skin cancers (NMSCs) [113]. Using tissue microarrays from 97 BCC and 105 cSCC cases, the authors demonstrated that cSCC exhibits significantly higher expression of CD4, CD8, Foxp3 (regulatory T cells), CD68, CD163, and CD11c across both the tumor core and invasive front compared to BCC, with the invasive front showing the most pronounced immune cell accumulation in both entities. Notably, cSCC displayed elevated Foxp3/CD8 and Foxp3/CD4 ratios, indicating greater regulatory T-cell dominance, while both tumor types showed M2-like macrophage predominance (CD163+/CD68+), with BCC exhibiting a more pronounced M2 polarization when comparing the CD163/CD68 and CD163/CD11c ratios. The study identified clinicopathological associations in cSCC including CD4 expression with T1 status, CD11c with nodal involvement, CD68 with infiltration depth, and CD8/CD11c with previous tumor history [113]. These findings suggest that immune profiling of NMSC could enable patient stratification for immunotherapy, as the observed differences in the immune landscape between BCC and cSCC and the variable responses to checkpoint inhibitors (51% response rate in cSCC, 6–31% in BCC) underscore the need for biomarker-driven treatment selection rather than a one-size-fits-all approach, potentially explaining why nearly half of advanced cSCC patients remain unresponsive to current PD-1-based immunotherapies [113].

#### 5.2.3. Tumor Mutational Burden

TMB measures somatic mutations per megabase of the specific tumor genome [114]. TMB has limited application as a biomarker for cSCC; however, its role as a predictor of response to immunotherapy has been studied [115]. A major limitation to the TMB developing into a robust biomarker is the lack of predictive cut-offs and heterogeneity in the methods used to study TMB across different studies [116]. Standardization of the cut-offs of different panels to measure TMB is needed for the clinical applicability of the TMB as a potential biomarker in cSCC along with PD-L1.

Commercially available GEP-40 test has the greatest impact on risk stratification to tailor individualized management and surveillance paradigms.

## 6. Application of AI in Precision Diagnostics in cSCC

### 6.1. AI-Assisted Histopathology

Deep learning models applied to H&E-stained whole-slide images can achieve dermatopathologist-level accuracy for distinguishing invasive SCC from benign mimickers, grading differentiation, and flagging perineural or lymphovascular invasion [117,118] (Table 4). Beyond binary classification, emerging systems provide heatmaps and feature attribution to highlight suspicious regions, support quality assurance, and enable large-scale, standardized scoring across institutions [117,118]. Currently, an important limitation of AI algorithms is the use of a training dataset from a single institution or small cohorts. Variations in scanner models also lead to heterogeneity in the pooled data. Thus, well-curated reference datasets from different cancer centers could possibly aid in standardizing AI algorithms in the future. [77] Additionally these datasets will need continual validation to improve accuracy.

### 6.2. Multinomic Integration and Precision Imaging

Integrative frameworks that combine genomics, transcriptomics, proteomics, radiomics, and advanced imaging are beginning to define molecularly and morphologically coherent cSCC subtypes [119]. In epithelial cancers, single-cell and spatial technologies can reveal intratumoral heterogeneity, therapy-tolerant clones, and immune niches, which can be linked to imaging phenotypes and digital pathology features for more refined risk models and treatment selection [119,120].

Future management of cSCC will shift toward precision oncology, where treatment is guided by real-time genomic and clonal mapping rather than stage alone, especially in advanced cSCC. Single-cell sequencing approaches such as the Multi-Patient-Targeted (MPT) sequencing method will enable clinicians to identify key driver mutations, detect rare aggressive subclones early, and monitor tumor evolution to anticipate resistance or metastasis. This will support highly personalized therapy that targets both dominant and emerging clones, ultimately transforming care from reactive treatment to proactive interception of high-risk tumor trajectories [121].

### 6.3. Label-Free Optical Tools and Machine Learning

Label-free optical platforms, such as Raman spectroscopy integrated with optical coherence tomography (Raman-OCT), can provide biochemical and microstructural contrast without exogenous dyes, enabling real-time, in vivo discrimination of SCC from normal skin [122]. When coupled with machine-learning classifiers trained on spectral–morphologic signatures, these systems achieve high accuracy for SCC diagnosis and margin assessment, offering a potential noninvasive complement or adjunct to conventional biopsy in precision cSCC workflows [122].

AI-driven quantitative histopathological features combined with traditional assessment modalities have several applications in the diagnostic algorithms of cSCC (Table 3). Currently, there are several challenges in the implementation of AI, as high-quality datasets lack generalizability. As these applications are being refined, further research is focusing on expanded training datasets, standardizing protocols and creating tailored guidelines based on AI-generated data [123,124].

The integration of radiomics and AI with traditional diagnostic tools such as histopathology along with optical imaging modalities including RCM or Raman spectroscopy have the potential to improve diagnostic workflows.

## 7. Conclusions

Precision diagnostics hold promise for optimizing outcomes in cSCC by shifting from reactive management to proactive, individualized care that minimizes overtreatment and maximizes survival. By fusing genomic profiling with digital pathology and AI-assisted feature extraction, alongside clinicopathologic hallmarks such as perineural invasion and differentiation grade, clinicians can delineate molecularly coherent subtypes of cSCC. Integrating molecular, digital, and clinicopathologic data has the potential to overcome the limitations of traditional staging systems, such as the AJCC 8th edition, which suffer from interobserver variability and incomplete prognostic stratification. This multimodal synthesis, enhanced by radiomics and spatial transcriptomics has the potential to enable more accurate risk models that transcend conventional histopathology. Further, ctDNA-based liquid biosignatures offer real-time, minimally invasive surveillance for recurrence and minimal residual disease.

However, the clinical applicability of the newer technologies is evolving. Although several modalities such as GEP-40 test, RCM, and hand-held MPM devices are FDA approved and commercially available, their widespread adoption is limited. Multi-institutional prospective trials must validate these technologies against cost-effectiveness benchmarks, addressing challenges such as data interoperability, regulatory hurdles for AI platforms, and equitable access to liquid biopsy infrastructure before their widespread application. Harmonized protocols for omics integration and AI training datasets will be essential to minimize bias and ensure reproducibility across diverse populations.

## Figures and Tables

**Table 1:** Key genetic alterations in cSCC.

Genetic Alteration	Frequency	Clinical Correlate
*TP53*	>50%	Altered DNA repair, genomic instability, poor disease-free survival
*CDKN2A*	25–50%	Dysregulation of the cell cycle, poor differentiation, metastasis
*NOTCH1*/*NOTCH2*	15–40%	Immune evasion, NOTCH signaling pathway
*FAT1*	5–20%	EMT upregulation, invasion and metastasis, poor differentiation
*RAS*	13–25%	UVB signatures, perineural invasion

**Table 2:** Common optical imaging techniques.

Technique	Mechanism	Depth	Strength	Limitation	Clinical Application
Dermatoscopy	Light microscopy	Epidermis–upper dermis	Rapid, cost-effective, widely available	Operator dependent, limited depth	Lesion screening
RCM	Infrared light (830 nm)	~200–300 μm	Noninvasive, real-time imaging	Limited depth, learning curve	In vivo diagnosis
MPM	Photon excitation (700–1000 nm)	~500–700 μm	Intrinsic collagen contrast, deep penetration, tumor–stroma interface evaluation	Longer acquisition time, limited availability	Assessment of invasion
EVCM	Reflectance imaging		Margin assessment on fresh unfixed tissue	Availability	Intraoperative margin assessment
Raman Spectroscopy	Light scattering	Superficial	High chemical specificity	Limited penetration, slow acquisition	Molecular characterization

RCM—Reflectance confocal microscopy; MPM—Multiphoton microscopy; EVCM—Ex vivo confocal microscopy.

**Table 3:** Liquid biopsy biomarkers in cSCC.

Biomarker	Strength	Limitation	Current State in cSCC
Circulating tumor DNA (ctDNA)	High specificity, quantitative monitoring and detection of minimal residual disease and/or early recurrence	Low sensitivity with low tumor burden, no standardization of cut-off values	Ongoing clinical trials evaluating clinical utility. Currently not FDA approved for diagnosis of cSCC
Circulating tumor cells (CTCs)	Intact tumor cells, potential to use in single-cell sequencing	Isolation techniques vary, difficult to trace primary cancer in cases of multiple cSCCs	Correlation with metastatic cSCC and nodal disease. Currently, the implementation of CTCs in cSCC is still investigational
Circulating microRNAs (miRNAs)	Measured with commonly available PCR-based assays, promise as diagnostic and prognostic signatures for potential use in longitudinal monitoring	Heterogeneity in reproducibility across all cohorts, standardization of assays is a challenge	Not approved for clinical use, larger validation and prospective studies needed before routine use
Proteomic plasma profiling (PPP)	Isolation of proteins from blood has high sensitivity and standardization	Stability of proteins is an issue	More reports on proteins in cSCC needed, possibly a composite marker of ctDNA, and PPP may be a better biomarker in the future

**Table 4:** Applications of artificial intelligence (AI) in precision diagnosis of cSCC.

AI Application Area	AI Workflow	Performance Metrics	Clinical Application
Automated clinical image classification	Analyze dermoscopic or clinical images	Accuracy 98.6% Sensitivity 98.3% Specificity 98.9% Precision 98.9%	Early detection, screening, remote settings
Digital pathology	Whole-slide image (WSI) analysis		Enhanced diagnostic accuracy, reproducibility, data storage
Margin assessment	AI-enhanced confocal microscopy, optical coherence tomography (OCT) segmentation models	Sensitivity 80% PPV 100% NPV 50%	Rapid noninvasive assessment
Radiomics	AI-analyzed imaging modalities such as US/CT/MRI		Staging accuracy, reduced unnecessary biopsies or further imaging
Clinical decision support systems (CDSS)	AI-supported decision tree learning models		Improves standardization of care and adherence to guidelines (such as the NCCN)

AI—artificial intelligence; US—ultrasound; CT—computed tomography; MRI—magnetic resonance imaging; PPV—positive predictive value; NPV—negative predictive value; NCCN—National Comprehensive Cancer Network.

## Data Availability

No new data were created or analyzed in this study.
